# A Cornflower Extract Containing *N*-Feruloylserotonin Reduces Inflammation in Human Skin by Neutralizing CCL17 and CCL22 and Inhibiting COX-2 and 5-LOX

**DOI:** 10.1155/2021/6652791

**Published:** 2021-09-13

**Authors:** Christophe Carola, Andrew Salazar, Christin Rakers, Franck Himbert, Quoc-Tuan Do, Philippe Bernard, Joerg von Hagen

**Affiliations:** ^1^Merck KGaA, Frankfurter Str. 250, 64293 Darmstadt, Germany; ^2^Merck Healthcare KGaA, Frankfurter Str. 250, 64293 Darmstadt, Germany; ^3^Greenpharma, 3 Allée du Titane, 45000 Orléans, France

## Abstract

Thymus and Activation-Regulated Chemokine (TARC/CCL17) and Macrophage-Derived Chemokine (MDC/CCL22) are two key chemokines exerting their biological effect *via* binding and activating a common receptor CCR4, expressed at the surface of type 2 helper T (Th2) cells. By recruiting Th2 cells in the dermis, CCL17 and CCL22 promote the development of inflammation in atopic skin. The aim of this research was to develop a plant extract whose biological properties, when applied topically, could be beneficial for people with atopic-prone skin. The strategy which was followed consisted in identifying ligands able to neutralize the biological activity of CCL17 and CCL22. Thus, an *in silico* molecular modeling and a generic screening assay were developed to screen natural molecules binding and blocking these two chemokines. *N*-Feruloylserotonin was identified as a neutraligand of CCL22 in these experiments. A cornflower extract containing *N*-feruloylserotonin was selected for further *in vitro* tests: the gene expression modulation of inflammation biomarkers induced by CCL17 or CCL22 in the presence or absence of this extract was assessed in the HaCaT keratinocyte cell line. Additionally, the same cornflower extract in another vehicle was evaluated in parallel with *N*-feruloylserotonin for cyclooxygenase-2 (COX-2) and 5-lipoxygenase (5-LOX) enzymatic cellular inhibition. The cornflower extract was shown to neutralize the two chemokines *in vitro*, inhibited COX-2 and 5-LOX, and demonstrated anti-inflammatory activities due mainly to the presence of *N*-feruloylserotonin. Although these findings would need to be confirmed in an *in vivo* study, the *in vitro* studies lay the foundation to explain the benefits of the cornflower extract when applied topically to individuals with atopic-prone skin.

## 1. Introduction

Up to 40% of the general population reports to have sensitive skin, and around 9% even have very sensitive skin [1, 2]. Sensitive skin encompasses increased skin sensitivity, increased skin irritability, and atopic-prone skin.

Atopic dermatitis (AD) is a chronic inflammation of the skin associated with elevated serum immunoglobulin E (IgE) levels [3]. Histologically, AD is characterized by infiltration of T-cells, monocytes, and eosinophils in the dermis of lesional skin [4]. Predispositions to these characteristics are observed in atopic-prone skin. One of the predominant physiologic hypotheses regarding atopic skin implies the dysfunction of the natural skin barrier [5]. Environmental factors such as viruses, allergens, or chemicals trigger the secretion of the cytokine thymic stromal lymphopoietin (TSLP) in the keratinocytes, the major cell types of epidermis [6]. After exposure to TSLP, dendritic cells migrate to near lymph nodes to trigger the proliferation of the naive CD4+ T-cells and their subsequent differentiation into type 2 helper T (Th2) cells [7]. TSLP is also capable of directly promoting Th2 cell differentiation of naive T-cells [8]. Following exposure to this cytokine, the dendritic cells in the skin lesions secrete two chemoattractive small chemokines: Thymus and Activation-Regulated Chemokine (TARC/CCL17) [9] and Macrophage-Derived Chemokine (MDC/CCL22) [10]. CCL17 and CCL22 are known to promote the development of skin inflammation in particular in atopic skin conditions [11, 12]. These two cytokines exert their biological effects through binding and activating the receptor CCR4, expressed on the surface of Th2 [13]. By recruiting Th2 cells at the site of inflammation, CCL17 and CCL22 promote further proinflammatory activities by the Th2 secretion of allergy-promoting cytokines like interleukin-4 (IL-4), interleukin-5 (IL-5), and interleukin-13 (IL-13) responsible for the promotion of immunoglobulin E (IgE) production by B cells [14]. IgE induces the activation of mast cells, leading to the secretion of additional Th2 cytokines [15]. Th2 cells are crucial for the initiation of the acute phase of the skin inflammation process before a switch from Th2 response to Th1 response promotes the chronic phase of atopic dermatitis [14].

Leukotrienes and prostaglandins are lipid signaling mediators of the arachidonic pathway known to play a crucial role in inflammation [16]. Biosynthesis of leukotrienes starts with the production of leukotriene A_4_ (LTA_4_), resulting from the metabolization of arachidonic acid by the enzyme 5-lipooxygenase (5-LOX). LTA_4_ is enzymatically converted into the leukotriene LTB_4_ (among other leukotrienes) [17].

Prostaglandins are the products of the conversion of arachidonic acid *via* the cyclooxygenase (COX) enzymes. COX-1 is constitutively expressed in most tissues, whereas COX-2 is induced in response to inflammatory stimuli [18]. Both enzymes convert arachidonic acid into prostaglandin PGH_2_. PGH_2_ is further converted by specific synthases into prostaglandins like PGE_2_ or PGD_2_.

LTB_4_ is involved in the recruitment of neutrophils and Th2 cells in the atopic dermatitis skin lesions [19] as these cells express BLT_1_, a high-affinity receptor of LTB_4_ [20]. PGE_2_ has been reported to promote the production of CCL17 in human dendritic cells *in vitro* [21, 22]. PGE_2_ was also shown in an *in vitro* study to inhibit the production of filaggrin in keratinocytes implying its role in the deteriorating barrier function in atopic dermatitis [23]. The prostaglandin PGD_2_ is present at high concentration in patients with atopic dermatitis and is the main prostaglandin produced by mast cells [24]. PGD_2_ is key in recruiting Th2 cells, eosinophils, and basophils, triggering an inflammatory cascade in atopic dermatitis [25].

*Centaurea cyanus*, also called cornflower, is native to Europe and belongs to the *Asteraceae* family plant. The flowers have an intense blue color, produced in the flowerheads, due to the blue pigment protocyanin [26]. A multistep extraction including a supercritical CO_2_ extraction step of this flower afforded an extract, in which the key bioactive ingredient component is *N*-feruloylserotonin (*N*-feruloyl-5-hydroxytryptamine, MW = 352.4). The content of *N*-feruloylserotonin in this extract varies between 3% and 8% (*w*/*w*).

*N*-Feruloylserotonin commonly known as Moschamine is a natural substance arising from the amide formation of a phenylpropanoid acid (feruloyl acid) and a tryptamine (serotonin). This phytochemical belongs to the group of safflomide-type phenylpropanoid acid amides and was first isolated from *Centaurea moschata* in 1997 [27]. 4 years later, Sarker et al. [28] isolated it from *Centaurea cyanus*.

A mixture of a cornflower extract and maltodextrin called CFEM is titrated to 1% of *N*-feruloylserotonin [29]. With the pure extract being very hygroscopic, the addition of maltodextrin helps maintain a free-flowing powder characteristic. CFEM is intended to be incorporated into an emulsion for topical applications.

The aim of the research presented in this paper was to develop a plant extract whose biological properties could be beneficial for individuals with atopic-prone skin conditions. The strategy which was followed consisted in identifying ligands able to neutralize the biological activity of CCL17 and/or CCL22, two key chemokines known to promote the development of skin inflammation in atopic skin conditions [11, 12]. The neutralization of these chemokines would limit Th2 chemotaxis in the skin and hence reduce acute and chronic inflammation in the skin.

A molecular modeling and a generic screening assay were developed to identify ligands of CCL17 and/or CCL22 from a pool of small natural molecules. This assay was designed to distinguish ligands blocking the receptor CCR4 (receptor antagonists) from those blocking the chemokine (neutraligands) [30]. *N*-Feruloylserotonin was identified as a neutraligand of CCL22 in this experiment.

A cornflower extract containing *N*-feruloylserotonin as the main component was selected for further *in vitro* tests: the gene expression modulation of inflammation biomarkers induced by CCL17 or CCL22 in the presence or absence of this extract was assessed in a keratinocyte cell line (HaCaT). This cornflower extract mixed to maltodextrin (CFEM) was evaluated in parallel with *N*-feruloylserotonin for COX-2 and 5-LOX enzymatic cellular inhibition.

The cornflower extract showed anti-inflammatory activities *in vitro* mainly due to the presence of *N*-feruloylserotonin with potential applications in the framework of atopic-prone skin conditions.

## 2. Material and Methods

### 2.1. *In Silico* Studies: Molecular Modeling of CCL17 and CCL22

Molecular modeling was performed with Sybyl-X 2.1.1 (Certara) except otherwise mentioned.

#### 2.1.1. CCL22 Homology Modeling

CCL17 was chosen as a template structure to design the CCL22 model. Human CCL17 protein structure is available in PDB (PDB code:1NR4). Human CCL17 and CCL22 primary sequences were retrieved from http://www.uniprot.org (accession number: Q92583-CCL17_HUMAN and O00626-CCL22_HUMAN, respectively). ClustalW as implemented on the server NPSA was used for sequence alignment. Then, the module Orchestrar of the Sybyl package was used to build the CCL22 3D structure based on CCL17 (parameters are default one: find and build the conserved regions of chemokines, add loops, model side chains, and minimize the internal energy of the modeled structure to release constraints).

#### 2.1.2. Docking Studies

Docking studies were performed with Surflex-dock51 as implemented in the Sybyl package. The default parameters and docking mode “GeomX” to obtain 20 conformations for each neutraligand were applied. The “protomol,” defining the docking site, was used accordingly to identify binding sites.

### 2.2. *In Vitro* Studies: Identification of Neutraligands of CCL17 and CCL22

Construction of a Coexpressing Gqi5 and CCR4 Cell Line [30]: Human Gqi5 cDNA was produced by PCR-based site-directed mutagenesis of the last 5 amino acids of Gq into Gi using the forward primer 5′-CCTCCAGTTGAACCTGAAGGACTGCGGCCTCTTCT AACTCGAGTCTAGAGGGC-3′ and the reverse primer 3′-GCCCTCTAGAC TCGAGTTAGAAGAGGCCG CAGTCCTTCAGGTTCAACTGGAGG-5′. Gqi5 cDNA was cloned into pcDNA 3.1 plasmid (Invitrogen) by overlap extension method. EGFP-CCR4-HEK cells were transiently transfected with the plasmid encoding Gqi5. The experiments were carried out 24 h after transfection to allow for Gqi5 protein expression.

### 2.3. Ligand Binding Assay

For cell plate preparation, EGFP-CCR4+Gqi5+HEK cells were loaded with fluorescent Ca^2+^-indicator Indo-1AM (5 *μ*M, Interchim) for 45 min at 37°C. The cells were detached in PBS-EDTA (5 mM) for 2 min, suspended in growth medium, pelleted by centrifugation at 320 × g for 5 min, and resuspended in HEPES-BSA buffer (137.5 mM NaCl, 6 mM KCl, 1.25 mM CaCl_2_, 1.25 mM MgCl2, 0.4 mM NaH_2_PO_4_, 5.6 mM glucose, 10 mM HEPES, BSA (0.1%, *w*/*v*), pH 7.4). Cells (100,000 cells/well, 100 *μ*L) were seeded in black clear bottom 96-well plates (Greiner) and centrifuged at 250 × g for 5 min.

#### 2.3.1. Ligand Plate Preparation

Two plates (96-well plates, Greiner) were prepared for the screening: one containing test compounds in order to determine their direct agonistic activity and a second containing test compounds mixed with CCL17 or CCL22 at room temperature for 1 h (neutraligand protocol). The chemokines and test compounds were prepared at a 3x concentration, while digitonin was prepared at a 4x concentration. The CCR4 antagonist, C-021 dihydrochloride 3581 [31], served as control in the experiment. In the antagonist protocol, compounds were added to the cell plate (one compound per well) and incubated with cells for 30 min at room temperature, while the agonist (CCL17 or CCL22) was added to the ligand plate only.

#### 2.3.2. Recording Calcium Responses in Indo-1- (a Fluorescent Calcium Indicator) Loaded CCR4+ Gqi5+ HEK Cells

Dye-loaded cells were placed in the FlexStation 3 (Molecular Devices). Calcium mobilization was measured at 401 and 475 nm emission fluorescence after excitation at 355 nm. For the primary screening, the ligands (50 *μ*L of chemokine mixed with DMSO or test compounds) were added to the cells after 40 s, followed by digitonin (50 *μ*L) after additional 60 s. Molecules were screened at a final concentration of 2 *μ*g/mL with 5 nM CCL17 or CCL22. DMSO final concentration did not exceed 1% on cells. Raw fluorescence data (at 401 and 475 nm) were exported, and the ratio 401/475 was calculated. The calcium response values (height of peak response over baseline) were determined. Hits were selected by comparing the response amplitude at the time of the chemokine addition ± test compound.

Hit molecules were confirmed by testing concentration-response, and the half maximal inhibitory concentration (IC50) values were calculated using the KaleidaGraph software (synergy).

### 2.4. Identification of the Main UV-Absorbing Peaks of the Cornflower Extract

The cornflower extract obtained after a multistep extraction process was studied by liquid chromatography coupled with a High-Resolution Mass Spectrometry maXis QqTOF (Bruker). Liquid chromatography was carried out on an UHPLC Ultimate 3000 (Thermo Scientific) with a Luna Omega column PS 1.6 *μ*m (150 × 2.1 mm), at 40°C with a flow of 300 *μ*L/min (see Table 1).

The three major compounds of the chromatographic profiles were characterized by retention time, UV maximum, monoisotopic mass, and fragmentation in MS^2^. All these data led to the unambiguous identification of each peak.

### 2.5. Evaluation of the Gene Expression Modulation of CCL26, MMP-2, IL-18, and CCL5 in the Presence of the Cornflower Extract in HaCaT Cells

The immortalized human keratinocyte cell line HaCaT (DKFZ, Heidelberg, Germany) was cultured in Dulbecco's Modified Eagle Medium (DMEM) (Invitrogen) supplemented with 10% fetal bovine serum (FBS) (Gibco-BRL), 100 U/mL penicillin (Invitrogen), 100 *μ*g/mL streptomycin (Invitrogen), and 2 mM L-glutamine (Invitrogen) at 37°C and 5% CO_2_. HaCaT cells were seeded in a 10 cm^2^ plate and grew until confluence (6 × 10^6^ cells). Cells were washed twice with PBS and were treated with culture medium in the absence or presence of chemokine (100 ng/mL CCL22 or CCL17) preincubated with DMSO or with the cornflower extract (1 mg/mL) titrated to 5% of *N*-feruloylserotonin and additionally without chemokines. Once treated, the cells were incubated, and the mRNA expression was quantified after 24 h. Total RNA was isolated from HaCaT cells, homogenized in TRI reagent (Euromedex), and purified using a RNeasy mini kit (Qiagen) according to the manufacturer's protocol. 1 *μ*g of total RNA was reverse transcribed using the MMLV Reverse Transcription Reagents (Invitrogen), and cDNA was amplified by PCR using TaqMan Universal PCR Master Mix (Applied Biosystems) on an ABI Prism 7700 Sequence detector with the following probes (Life Technologies): ribosomal 18S (cat no. Hs99999901_s1), IL-18 (cat no. Hs01038788_m1), CCL26 (cat no. Hs00171146_m1), MMP2 (cat no. Hs01548727_m1), and CCL5 (cat no. Hs00982282_m1).

PCR conditions were as follows: 50°C (2 min), 95°C (10 min) followed by 45 amplification cycles of 95°C (15 s), and 60°C (1 min).

Relative gene expression was calculated using the delta-delta CT method, after normalization to ribosomal 18S expression. Experiments were performed in triplicate.

### 2.6. Molecular Modeling of COX-2

The 3D protein structure of human COX-2 (Prostaglandin G/H synthase 2, Uniprot ID P35354) cocrystallised with rofecoxib (Vioxx) was obtained from Protein Data Bank entry 5KIR [32]. The structure was prepared according to the default protein preparation protocol of the Protein Preparation Wizard by Schrödinger [33] including H-bond optimization at pH 7.0 and restrained protein structure minimization with force field OPLS3e [34]. Glide [35] was used to perform induced fit docking simulations of *N*-feruloylserotonin and COX-2 [36].

### 2.7. *In Vitro* Studies: Inflammatory Enzyme Inhibition

#### 2.7.1. COX-2 Enzymatic Activity Assay

COX-2 enzymatic activity was assayed using a COX-2 activity assay kit (BioVision, USA) as per the manufacturer's instructions. Briefly, exponentially growing THP-1 monocytes in RPMI media supplemented with 10% FCS were seeded at a density of 5 × 10^5^ cells/mL and differentiated to macrophages by stimulating with 200 ng/mL PMA for 48 hours and incubated at 37°C, 5% CO_2_. The macrophages were pretreated with either the product CFEM (0.01%) or the *N*-feruloylserotonin (0.0001%) for 1 hour. For COX-2 induction, 1 *μ*g/mL LPS was added to the medium with test substance for 24 hours. The cells were incubated at 37°C. After incubation, the cells were harvested and washed in ice-cold PBS (Biochrom, Merck, Germany). The Path Scan Sandwich ELISA Lysis Buffer (Cell Signaling Technology, USA) was used to generate a cell lysate which was further used in the assay. Protein concentration of the lysate was determined spectrophotometrically *via* a NanoDrop One spectrophotometer (Thermo Fischer Scientific, Germany). The assay was read spectrophotometrically at 280 nm with a Tecan Spark 20M (Tecan, Switzerland) at Ex/Em 535/590 nm, and the values of the samples were evaluated by comparison to a resorufin standard curve. A recombinant COX-2 enzyme (Merck, Germany) was used as a positive control as this recombinant enzyme was inhibited by celecoxib (BioVision, USA) as negative control. COX-2 enzymatic activity was calculated as per the manufacturer's instructions.

The concentrations tested were determined to be noncytotoxic by a previously performed ATPlite 1step Luminescence Assay (PerkinElmer, USA) that assessed cellular viability via ATP.

#### 2.7.2. 5-LOX Enzymatic Activity Assay

LOX enzymatic activity was assayed using a LOX activity assay kit (BioVision, USA) as per the manufacturer's instructions. Exponentially growing THP-1 monocytes in RPMI media supplemented with 10% FCS were seeded at a density of 4 × 10^5^ cells/mL. These monocytes were pretreated with the test substances for 1 hour at 37°C, 5% CO_2_. For 5-LOX induction, 10 ng/mL IL-3 was added to the medium with test substance for 24 hours. The cells were incubated at 37°C. After incubation, the cells were harvested and washed in ice-cold PBS (Biochrom, Merck, Germany). The Path Scan Sandwich ELISA Lysis Buffer (Cell Signaling Technology, USA) was used to generate a cell lysate which was further used in the assay. Protein concentration of the lysate was determined spectrophotometrically at 280 nm *via* a NanoDrop One spectrophotometer (Thermo Fischer Scientific, Germany). The assay was read spectrophotometrically with a Tecan Spark 20M (Tecan, Switzerland) at Ex/Em 485/535 nm, and the values of the samples were evaluated by comparison to the standard curve of the supplied probe standard. The supplied LOX inhibitor (BioVision, USA) was used as a negative control. LOX enzymatic activity was calculated as per the manufacturer's instructions. The concentrations tested were determined to be noncytotoxic by a previously performed ATPlite 1 step Luminescence Assay (PerkinElmer, USA) that assessed cellular viability *via* ATP.

## 3. Results and Discussion

CCL17 and CCL22 are key chemokines in atopic dermatitis: the serum levels of these chemokines were found to be elevated in AD patients and proportional to disease severity [37, 38]. These two cytokines exert their biological effects through binding and activating the receptor CCR4, expressed on the surface of T-helper 2 (Th2) [13]. By recruiting Th2 cells at the site of inflammation, CCL17 and CCL22 promote further proinflammatory activities by the Th2 secretion of allergy-promoting cytokines [14].

A classical drug development strategy to limit the extravasation of Th2 in the atopic-prone skin consists in identifying an antagonist of the receptor CCR4 to modulate the chemokine system signaling. Indeed, it was demonstrated that CCR4 played a key role in the induction of acute atopic dermatitis skin lesions in a skin allergic inflammation of BALB/c mice, a strain prone to Th2 response [39]. An efficient antagonist of CCR4 to inhibit Th2 infiltration and improve the AD-like skin lesions in the same BALB/c mouse model has been recently reported [40]. However, this receptor blockage strategy is challenging as receptor antagonists in general showed sometimes partial agonistic or inverse agonistic activities [41]. Some CCR4 antagonists were even reported to act as agonists for cell chemotaxis [42].

Nature has developed other original strategies based on chemokine blockade rather than receptor antagonists to prevent or control inflammation: pathogens and parasites express peptides [43] and soluble proteins [44, 45] that act as decoy molecules and prevent the chemokine to bind to their cognate receptors. Based on this bioinspiration approach, it was reported that two RNA aptamers could efficiently neutralize CCL17 in a murine model of contact hypersensitivity and hence inhibited T-cell chemotaxis in this *in vivo* model [46]. In another murine model of allergic airway hypereosinophilia, a synthetic substance with a pyrimidinone scaffold was shown to be a neutraligand of the chemokine CXCL12 with anti-inflammatory properties [47]. Another class of substance, an oligonucleotide, was also found to be a neutraligand of CXCL12. This oligonucleotide showed encouraging results in a recent phase IIa clinical study in patients with antichronic lymphocytic leukemia (CLL) in combination with anti-CLL drugs opening the door to further clinical developments [48].

In this work, a bioinspired approach was used to identify a natural substance to neutralize the chemokines CCL17 and CCL22, alternative to conventional inhibition of the receptor CCR4 at the surface of Th2 cells [30].

### 3.1. *In Silico* Docking of *N*-Feruloylserotonin in the Chemokines CCL17 and CCL22

**Figure 1:**
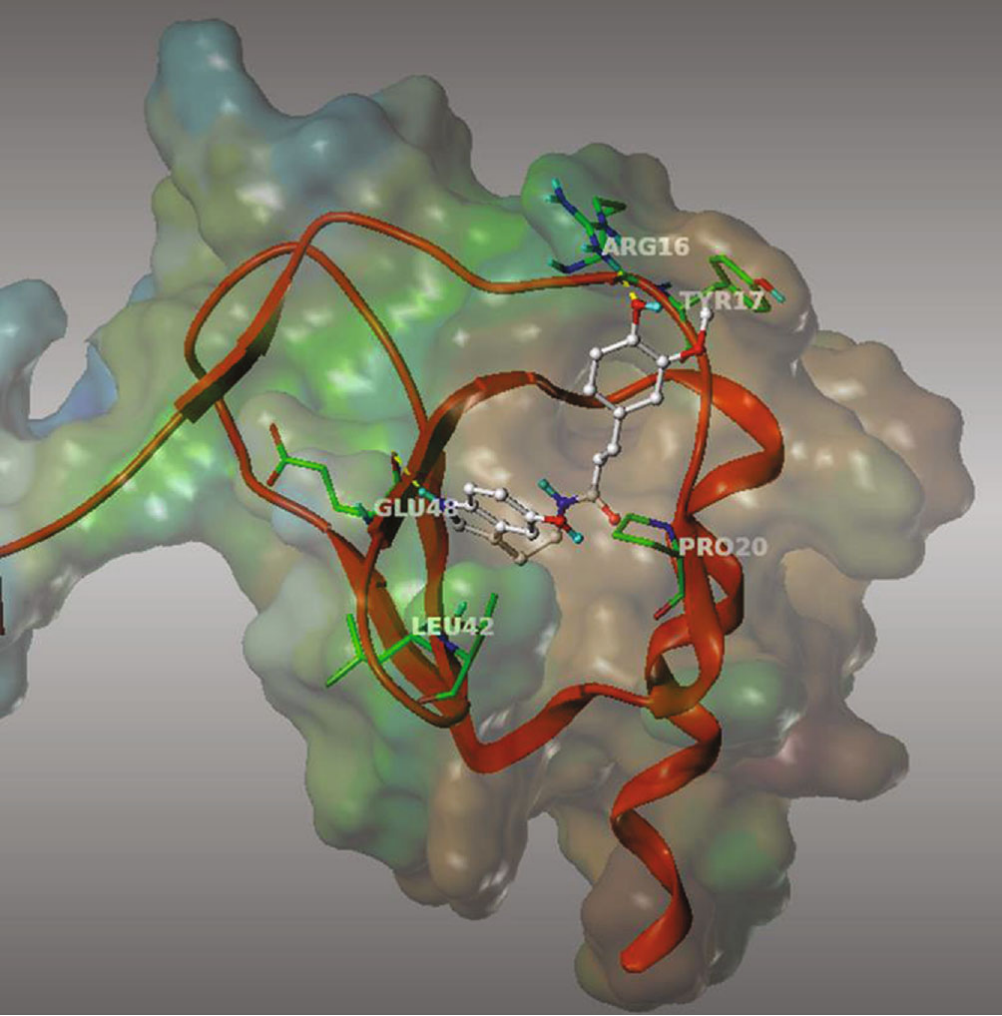
3D representation of the binding of *N*-feruloylserotonin to the chemokine CCL22.

A series of natural substances were screened in the molecular modeling program Sybyl-x 2.1.1 to evaluate their affinity on the structures of CCL17 and CCL22. The structure of CCL17 was available in the Protein Data Bank (PDB) which is not the case for CCL22. For this reason, the CCL22 structure was built, based on its sequence homology with the 3D structure of CCL17. Among the substances screened, the chemical structure of *N*-feruloylserotonin showed affinity to the structure of CCL22 only. An in-depth molecular docking study was carried out to determine the interactions between *N*-feruloylserotonin and the amino acid residues of the chemokine CCL22. According to this *in silico* evaluation, *N*-feruloylserotonin binds to the amino acid residues: arginine 16 (ARG16), tyrosine 17 (TYR17), proline 20 (PRO20), leucine 42 (LEU42), and glutamic acid 48 (GLU48) (see Figure 1).

### 3.2. Cellular Assay to Evaluate the Ligand Binding Affinity of *N*-Feruloylserotonin for the Chemokine CCL17 and CCL22

*N*-Feruloylserotonin was tested in a cellular assay to confirm the *in silico* predicted ligand binding affinity of *N*-feruloylserotonin for the chemokine CCL22. The screening assay “Time-Resolved Intracellular Calcium Recording” (TRIC-r) was exploited [30] and is based on the kinetic measurement of chemokine-induced calcium response. Depending on the incubation conditions, either with the chemokine CCL22 or with the receptor CCR4, the TRIC-r method led to the unambiguous distinction of neutraligands (docking to the chemokine and neutralizing its biological activity) from the receptor antagonist (docking to the receptor) [30]. *N*-Feruloylserotonin was confirmed to be a neutraligand of CCL22 in this assay with an IC_50_ of 15 *μ*M (36% of calcic response induced by CCL22 at a concentration of 10 *μ*M). *N*-Feruloylserotonin was found not to be a neutraligand of CCL17.

### 3.3. Analysis of the Main Natural UV-Absorbing Compounds in a Cornflower Extract

**Figure 2:**
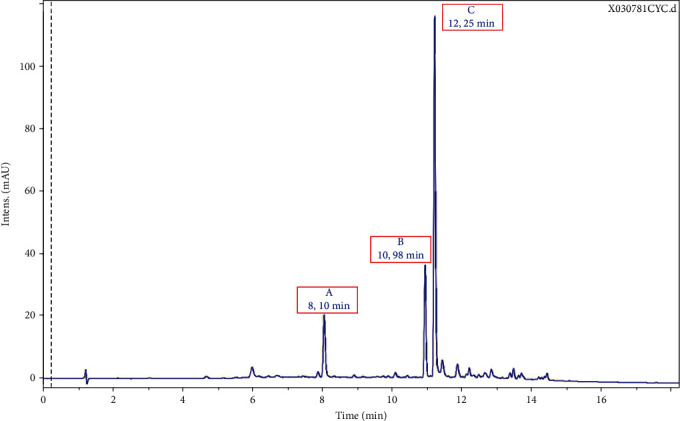
Chromatographic profile of a typical cornflower extract obtained from raw material after cold pressure in UV at 313 nm (concentration 0.5 mg/mL).

The cornflower was selected on the basis of its *N*-feruloylserotonin content [28]. The extraction process was designed to preserve the concentration of *N*-feruloylserotonin in the final extract. An analytical investigation was then performed to confirm the presence of this key substance and identify other UV-absorbing substances present in this extract. The LC-HRMS chromatographic profile of this extract was generated at a wavelength of 313 nm. The three major compounds which were studied are designated by capital letters A, B, and C (see Figure 2).

**Figure 3:**
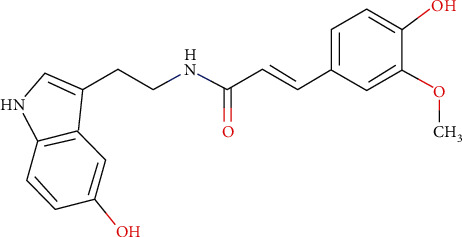
Molecular structure of *N*-feruloylserotonin (C_20_H_20_N_2_O_4_; MW = 352.4 g/mol).

Compound C, which is the main compound, is characterized by a monoisotopic mass of 352.1411 g/mol. As expected, this mass corresponds to *N*-feruloylserotonin (Figure 3) based on the literature relative to *Centaurea cyanus* [28]. In MS^2^, in the positive ionization mode, the ion of *m*/*z* 177.0539 resulting from the rupture of the amide function of the structure was identified. From this ion, the loss of the methyl from methoxy and of the hydroxy present on the structure was observed at *m*/*z* 145.0281 and finally the loss of the ketone at *m*/*z* 117.0330.

**Figure 4:**
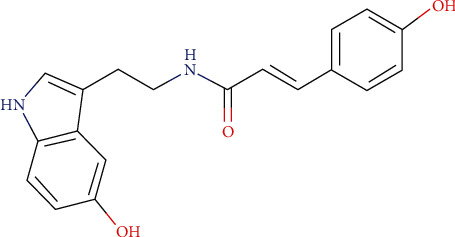
Molecular structure of *N*-p-coumaroylserotonin (C_19_H_18_N_2_O_3_; MW = 322.13 g/mol).

Compound B is characterized by a monoisotopic mass of 322.1309 g/mol. The Greenpharma database containing the analytical data of more than 300.000 natural substances was screened to identify this mass. Among the candidates that matched the monoisotopic mass of compound B, the only plausible structure was *N*-p-coumaroylserotonin (Figure 4).

By checking the fragmentation of this structure in MS^2^, the ion of *m*/*z* 147.0440 was observed and derives from the rupture of the amide function. Further degradation of this ion of *m*/*z* 147.0440 led to the loss of ketone (detected with the ion of *m*/*z* 119.0488). Structures of compounds B and C belong to the same structural family, and the observed fragmentation pathways are similar. Thus, compound B corresponds to *N*-p-coumaroylserotonin, a substance identified in 2 *Centaurea* species: *Centaurea nigra* [49] and *Centaurea vlachorum* [50] but not in *Centaurea cyanus*.

**Figure 5:**
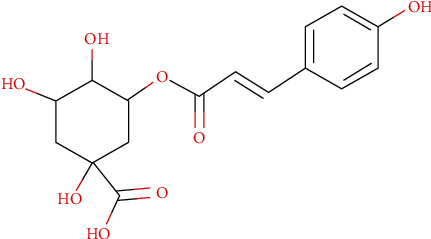
Molecular structure of p-coumaroylquinic acid (C_16_H_18_O_8_; MW = 338.31 g/mol).

Compound A is characterized by a monoisotopic mass of 338.0993 g/mol. Both modes of ionization (positive and negative) in MS led to a neutral loss of 146.036 corresponding to the monoisotopic mass of a deprotonated coumaroyl group. In MS^2^ negative, the ion of *m*/*z* 191.0554 corresponds to the fragmentation of p-coumaroylquinic acid (see Figure 5) after the neutral loss of the previously mentioned deprotonated coumaroyl group. Interestingly, this molecule which belongs to the chlorogenic acid family [51] had never been described in any organism of the genus *Centaurea*.

The main UV-absorbing substance in the cornflower extract is *N*-feruloylserotonin. The mass ratio of *N*-feruloylserotonin to *N*-p-coumaroylserotonin varies about 2.3 : 1, and the mass ratio of *N*-feruloylserotonin to p-coumaroylquinic acid varies about the ratio 6 : 1.

### 3.4. Evaluation of mRNA Expression (PCR-mRNA) of Inflammation Biomarkers in the Presence of the Cornflower Extract in a Cell Line Model

Upon being stimulated with interferon gamma (IFN-*γ*)/tumor necrosis factor alpha (TNF-*α*), keratinocytes can secrete chemokines, including CCL17 and CCL22 [52] which will induce atopic dermatitis-like inflammation conditions [53]. In this study, the identified CCL17 and CCL22 were added to the human keratinocyte cell line (HaCaT) to assure that the response is specifically attributed to these chemokines. The gene expression modulation of inflammation biomarkers induced by CCL17 or CCL22 in the presence or absence of a cornflower extract titrated to 5% of *N*-feruloylserotonin was tested in these cells. An initial treatment of HaCaT cells with CCL17 and CCL22 had been carried out, and several inflammatory regulated biomolecules, including interleukin-18 (IL-18), the chemokine CCL26, the chemokine CCL5, and the matrix metalloproteinase-2 (MMP-2), were found to be upregulated at the transcription level, monitored using RT-qPCR (data not shown). IL-18 is a cytokine known to be involved in the development of atopic diseases, especially atopic dermatitis [54], where CCL26 correlated with the severity of the disease [55]. In this ailment, CCL5 is responsible for the recruitment of leukocytes at the site of inflammation [56]. MMP-2 is a matrix metalloproteinase produced under inflammatory conditions and is involved in the infiltration of inflammation cells, in particular mast cells [57].

**Figure 6:**
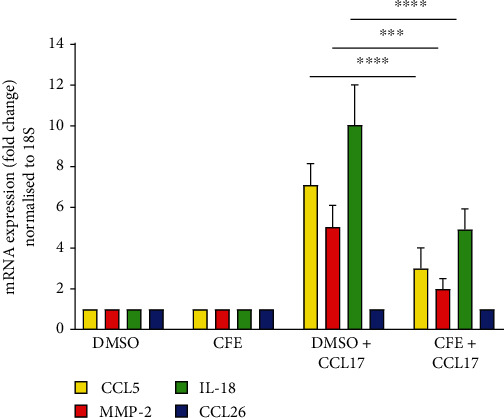
Effect of the pure cornflower extract (CFE, 1 mg/mL) on CCL5, MMP-2, and IL-8 expression after inflammation induction by CCL17. Relative gene expression was calculated using the delta CT method after normalization to ribosomal 18S expression. Experiment was performed in triplicate. One-way ANOVA: ^∗∗∗∗^*p* < 0.0001; ^∗∗∗^*p* < 0.005.

In this HaCaT cell line model, the treatment with the chemokine CCL17 upregulated 7-fold the gene expression of CCL5, 5.1-fold the gene expression of MMP-2, and 10.1-fold the one of IL-18. CCL17 did not show any effect on the gene expression of CCL26 *in vitro*. In the presence of cornflower extract (1 mg/mL), the treatment of the HaCaT cells with CCL17 upregulated the same previously mentioned biomarkers moderately: 3-fold for the gene expression of CCL5, 2-fold for the one of MMP-2, and 4.9-fold for the gene expression of IL-18 (see Figure 6).

**Figure 7:**
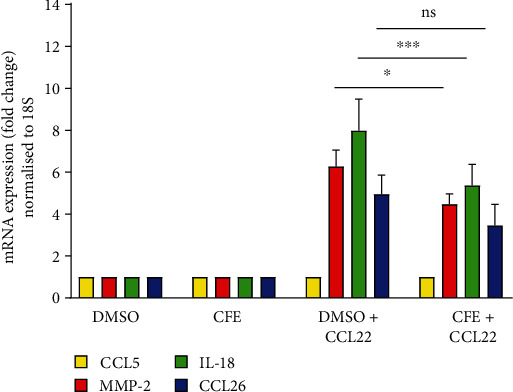
Effect of the pure cornflower extract (CFE, 1 mg/mL) on MMP-2, IL-8, and CCL26 expression after inflammation induction with CCL22. Relative gene expression was calculated using the delta-delta CT method after normalization to ribosomal 18S expression. Experiment was performed in triplicate. One-way ANOVA: ns: nonsignificant; ^∗^*p* < 0.05; ^∗∗∗^*p* < 0.005.

In HaCaT cells, the treatment with the chemokine CCL22 upregulated 6.3-fold the gene expression of MMP-2, 8-fold the gene expression of IL-18, and 4.9-fold the gene expression of CCL26. CCL22 did not affect the gene expression of CCL5. In the presence of the cornflower extract (1 mg/mL), after stimulation with CCL22, the upregulation of the gene expression of these cytokines was 4.5-fold for MMP-2, 5.4-fold for IL-18, and 3.5-fold for CCL26 (see Figure 7).

In both experiments, the cornflower extract significantly downregulated the gene expression of the measured inflammation biomarkers (CCL5, MMP-2, and IL-18 in the CCL17 experiment and MMP-2 and IL-18 in the CCL22 experiment) underlining its anti-inflammatory activity.

### 3.5. The Cornflower Extract Contains at Least One Neutraligand of CCL17

The downregulation of the expression of key proinflammatory biomarkers with the cornflower extract in the presence of CCL22 was expected. This result can be related to the presence of *N*-feruloylserotonin which is a neutraligand of CCL22, limiting its proinflammatory activity. However, the cornflower extract demonstrated an anti-inflammatory activity in the presence of CCL17 as well, although *N*-feruloylserotonin had not been identified *in silico* and *in vitro* as a neutraligand of this chemokine. Thus, the observed biological activity could likely be attributed to the presence of one or several other nonidentified substance(s) in the cornflower extract having a neutraligand activity towards CCL17. Interestingly, *N*-(p-coumaroyl)serotonin, the UV-absorbing component with the second highest concentration in the cornflower extract, was shown to exert a suppressive effect on proinflammatory cytokine production from monocytes underlining its anti-inflammatory activity [58, 59]. Consequently, this substance could also contribute to the anti-inflammatory potential of the cornflower extract and could be a candidate as neutraligand to CCL17, but further investigations are required to validate this hypothesis.

### 3.6. Proposed Binding of *N*-Feruloylserotonin to COX-2

**Figure 8:**
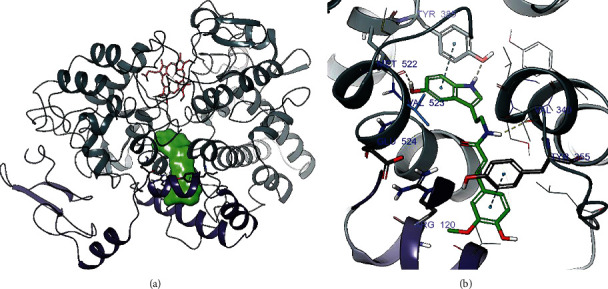
(a) Human COX-2 (PDB 5KIR) with docked *N*-feruloylserotonin in the active site shown as green surface. The heme group is depicted as red stick model. The three-pocket entry-restricting residues Arg120, Glu524, and Tyr355 surround the docked ligand as stick models. (b) Binding hypothesis of *N*-feruloylserotonin to COX-2 (docking score -11.41 kcal/mol). Protein-ligand interactions are indicated as blue dashed lines for aromatic interactions and yellow dashed lines for hydrogen bonds. Tyr385 has been shown to be actively involved in electron transfer between natural substrate arachidonic acid and heme [60].

The binding of *N*-feruloylserotonin to human COX-2 has been determined by computational docking that models amino acid residues flexibly thereby allowing conformational adaptation of the protein active site to the ligand. The binding mode shows protein ligand interactions (*π*-*π* stacking and hydrogen bonding) of the 5-hydroxyindole group of *N*-feruloylserotonin with catalytically important Tyr385 and the backbone of Met522. Further, the amide group establishes a hydrogen bond to the backbone of Val349 while the methoxyphenol part interacts with Tyr355 through *π*-*π* stacking. Tyr355, along with Arg120 and Glu524, is part of the amino acid ensemble that borders the ligand binding channel entry at the membrane binding domain of COX-2 (see Figure 8).

### 3.7. COX-2 Enzymatic Inhibition

**Figure 9:**
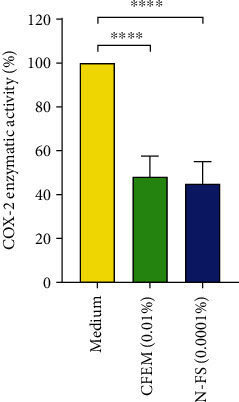
COX-2 enzymatic inhibition: endogenous COX-2 enzymatic activity in THP-1 cells differentiated in macrophages (PMA). Macrophages pretreated with CFEM or N-FS (N-feruloylserotonin) for 1 hour. COX-2 induction (LPS) with the test substance for 24 hours. One-way ANOVA: ^∗∗∗∗^*p* < 0.0001.

*N*-Feruloylserotonin was shown to exert influence on a number of biological pathways including anti-inflammatory pathways: it inhibits the enzymes COX-1 and COX-2 *in vitro* [61]. After triggering the differentiation of THP-1 monocytes to macrophages, the induction of COX-2 in cells was initiated with LPS. CFEM at a concentration of 0.01% yielded a significant inhibition of COX-2 activity in the cells by 52% in comparison to the control. *N*-Feruloylserotonin was tested at a concentration of 0.0001% (corresponds to the concentration in CFEM at 0.01%). *N*-Feruloylserotonin (0.0001%) showed a significant inhibition of COX-2 activity in the cells by 55%. Thus, the COX-2 inhibition of CFEM (containing 1% of *N*-feruloylserotonin) is mainly related to *N*-feruloylserotonin (see Figure 9). The *in silico* binding of *N*-feruloylserotonin in the active pocket of COX-2 and the reported enzymatic inhibition of COX-2 *in tubo* [61] confirm the biological activity of *N*-feruloylserotonin. Finally, *N*-feruloylserotonin was shown to inhibit the production of PGE_2_ in LPS-stimulated macrophages by downregulating the expression of COX-2 at the protein and mRNA levels [62].

### 3.8. 5-LOX Enzymatic Inhibition

**Figure 10:**
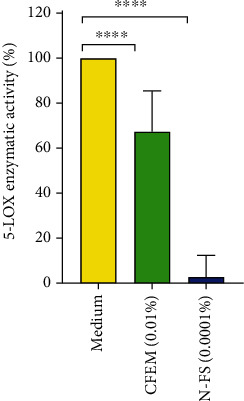
5-LOX enzymatic inhibition: endogenous 5-LOX enzymatic activity in THP-1 cells differentiated in macrophages (PMA). Macrophages pretreated with CFEM or N-FS (N-feruloylserotonin) for 1 hour. 5-LOX induction (IL-3) with the test substance for 24 hours. One-way ANOVA: ^∗∗∗^*p* < 0.005; ^∗∗∗∗^*p* < 0.0001.

After pretreatment of THP-1 monocytes with the test substances, the 5-LOX induction was induced with IL-3. CFEM (0.01%) yielded a significant inhibition of 5-LOX activity in cells by 23% in comparison to the control. *N*-Feruloylserotonin was tested at a concentration of 0.0001% (corresponds to the concentration in CFEM at 0.01%). *N*-Feruloylserotonin at 0.0001% showed a strong and significant inhibition of 5-LOX activity in cells by 97.3%. The 5-LOX inhibition of CFEM (containing 1% of *N*-feruloylserotonin) is partly related to the presence of *N*-feruloylserotonin in CFEM (see Figure 10).

### 3.9. The Prostaglandin/Leukotriene Signaling in Atopic-Prone Skin

5-Lipoxygenase (LOX-5) and cyclooxygenase-2 (COX-2) metabolize arachidonic acid into leukotrienes and prostaglandins, respectively [17]. Leukotrienes and prostaglandins are lipid signaling mediators of the arachidonic pathway known to play a crucial role in inflammation [16]. LTB_4_ is involved in the recruitment of Th2 cells in the atopic dermatitis skin lesions [19]. PGE_2_ promotes the production of CCL17 in human dendritic cells *in vitro* [21, 22] and inhibits the production of filaggrin in keratinocytes, underlining its role in the deteriorating barrier function in atopic dermatitis [23]. PGD_2_ is key in recruiting Th2 cells, eosinophils, and basophils, triggering an inflammatory cascade in atopic dermatitis [25]. CFEM was shown to be a dual COX-2/5-LOX inhibitor decreasing the production of the proinflammatory leukotriene LTB_4_ and of the two prostaglandins PGE_2_ and PGD_2_ in atopic-prone skin. As discussed in the previous sections, this dual biological activity can be linked to the presence of *N*-feruloylserotonin in the cornflower extract.

### 3.10. Mode of Action of CFEM and of *N*-Feruloylserotonin in Atopic-Prone Skin

**Figure 11:**
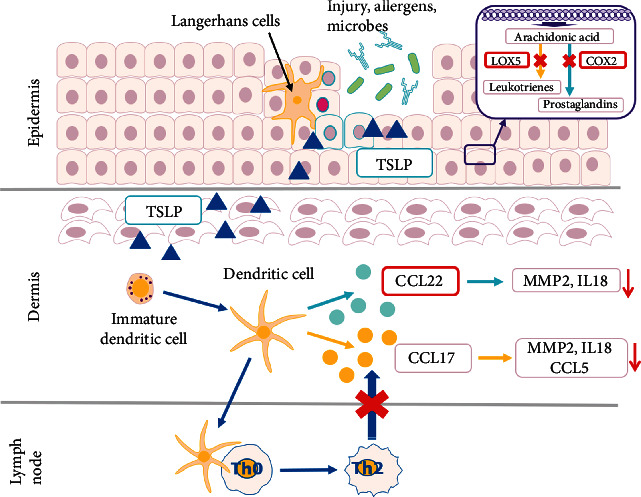
Mode of action of the cornflower extract (in red). The biological activities of *N*-feruloylserotonin are highlighted in red boxes.

Based on the results of this research, the mode of action of a topical application of a formulation containing the cornflower extract in the context of sensitive to atopic-prone skin could be summarized as follows: the cornflower extract could limit the recruitment of Th2 cells at the site of inflammation by neutralizing the chemokines CCL17 and CCL22 (thanks to the neutraligand potential of *N*-feruloylserotonin) and contributes to the reduction of acute and hence chronic inflammation in the skin. In parallel, the cornflower extract due to the presence of *N*-feruloylserotonin inhibits two key enzymes of the arachidonic pathway COX-2 and 5-LOX decreasing therefore the production of the proinflammatory leukotriene LTB_4_ and of the two prostaglandins PGE_2_ and PGD_2_ in atopic-prone skin (see Figure 11).

### 3.11. Serotonin-Like Activity of *N*-Feruloylserotonin

Atopic dermatitis is known as psychocutaneous disorder, and it is observed that psychotherapy of patients with atopic dermatitis improves the psychological and the dermatological status [63]. Kawana et al. [64] reported that tandospirone citrate, a serotonin receptor agonist of 5-HT1A, had a positive effect on the profile of mood states (POMS) in a clinical study in patients with atopic dermatitis and in parallel decreased itching. *N*-Feruloylserotonin was reported an agonist of the serotonin receptor type-1 (5-HT1) in renal tubular epithelial cells and was able to suppress AMP formation by binding to this receptor (negatively coupled to adenylate cyclase) [61]. This result indicates that *N*-feruloylserotonin could possess serotonergic activities. Based on these data, it can be hypothesized that *N*-feruloylserotonin acts in a similar way to tandospirone citrate and may have a positive effect on the anxiety score and therefore on the improvement of the skin condition in atopic dermatitis. Investigations need to be carried out to validate this hypothesis.

## 4. Conclusion

The anti-inflammatory activities of a cornflower extract titrated to 5% *N*-feruloylserotonin, of a mixture of the same extract titrated to 1% of *N*-feruloylserotonin in maltodextrin (CFEM), and of a pure sample of *N*-feruloylserotonin were evaluated in different *in vitro* biological models.

The presence of *N*-feruloylserotonin is essential to explain the anti-inflammatory activities of the cornflower extract. This natural compound was identified as a neutraligand of the chemokine CCL22, a chemokine which is upregulated in atopic-prone skin. Another nonidentified component of the cornflower extract neutralizes CCL17 which shares biological similarities to CCL22. By neutralizing CCL17 and CCL22, the cornflower extract limits the Th2 recruitment in the skin at the site of inflammation, reducing acute and hence chronic inflammation.

Additionally, it was observed that CFEM inhibited two key enzymes of the arachidonic pathway, 5-LOX and COX-2, preventing the formation of their respective proinflammation lipid signaling mediators LTB_4_ and PGE_2_ present at high concentration in atopic-prone skin. This can be linked to the presence of *N*-feruloylserotonin. Lastly, *N*-feruloylserotonin may even possess serotonin-like activities which could also contribute to the improvement of the atopic-prone skin condition.

These *in vitro* studies lay the foundation to explain the benefits of the cornflower extract when applied topically to individuals with atopic-prone skin. The next step will consist in the organization of a clinical study on volunteers with very sensitive skin to investigate the anti-inflammatory potential of a formulation of cornflower extract containing a defined amount of *N*-feruloylserotonin.

## Figures and Tables

**Table 1:** Elution gradient.

Time (min)	% formic acid (0.1% in water)	% formic acid (0.1% in methanol)
0	80	20
3	80	20
18	0	100

## Data Availability

Data are available on request.

## References

[1] Farage M. A. (2019). The prevalence of sensitive skin. *Frontiers in Medicine*.

[2] Misery L., Loser K., Stander S. (2016). Sensitive skin. *Journal of the European Academy of Dermatology and Venereology*.

[3] Leung D. Y. M. (2000). Atopic dermatitis: new insights and opportunities for therapeutic intervention. *The Journal of Allergy and Clinical Immunology*.

[4] Ong P. Y., Leung D. Y. (2006). Immune dysregulation in atopic dermatitis. *Current Allergy and Asthma Reports*.

[5] Seidenari S., Giusti G. (1995). Objective assessment of the skin of children affected by atopic dermatitis: a study of pH, capacitance and TEWL in eczematous and clinically uninvolved skin. *Acta Dermato-Venereologica*.

[6] Allakhverdi Z., Comeau M. R., Jessup H. K. (2007). Thymic stromal lymphopoietin is released by human epithelial cells in response to microbes, trauma, or inflammation and potently activates mast cells. *The Journal of Experimental Medicine*.

[7] He R., Oyoshi M. K., Garibyan L., Kumar L., Ziegler S. F., Geha R. S. (2008). TSLP acts on infiltrating effector T cells to drive allergic skin inflammation. *Proceedings of the National Academy of Sciences of the United States of America*.

[8] Indra A. K. (2013). Epidermal TSLP: a trigger factor for pathogenesis of atopic dermatitis. *Expert Review of Proteomics*.

[9] Imai T., Baba M., Nishimura M., Kakizaki M., Takagi S., Yoshie O. (1997). The T cell-directed CC chemokine TARC is a highly specific biological ligand for CC chemokine receptor 4. *The Journal of Biological Chemistry*.

[10] Mantovani A., Gray P. A., Van Damme J., Sozzani S. (2000). Macrophage-derived chemokine (MDC). *Journal of Leukocyte Biology*.

[11] Følsgaard N. V., Chawes B. L. K., Bønnelykke K., Jenmalm M. C., Bisgaard H. (2012). Cord blood Th2-related chemokine CCL22 levels associate with elevated total-IgE during preschool age. *Clinical & Experimental Allergy*.

[12] Saeki H., Tamaki K. (2006). Thymus and activation regulated chemokine (TARC)/CCL17 and skin diseases. *Journal of Dermatological Science*.

[13] Perros F., Hoogsteden H. C., Coyle A. J., Lambrecht B. N., Hammad H. (2009). Blockade of CCR4 in a humanized model of asthma reveals a critical role for DC-derived CCL17 and CCL22 in attracting Th2 cells and inducing airway inflammation. *Allergy*.

[14] Kim J. Y., Jeong M. S., Park M. K., Lee M. K., Seo S. J. (2014). Time-dependent progression from the acute to chronic phases in atopic dermatitis induced by epicutaneous allergen stimulation in NC/Nga mice. *Experimental Dermatology*.

[15] Kim T. H., Kim G. D., Jin Y. H., Park Y. S., Park C. S. (2012). Omega-3 fatty acid-derived mediator, Resolvin E1, ameliorates 2,4-dinitrofluorobenzene-induced atopic dermatitis in NC/Nga mice. *International Immunopharmacology*.

[16] Nicolaou A. (2013). Eicosanoids in skin inflammation. *Prostaglandins, Leukotrienes, and Essential Fatty Acids*.

[17] Honda T., Kabashima K. (2019). Prostanoids and leukotrienes in the pathophysiology of atopic dermatitis and psoriasis. *International Immunology*.

[18] Lee J. L., Mukhtar H., Bickers D. R., Kopelovich L., Athar M. (2003). Cyclooxygenases in the skin: pharmacological and toxicological implications. *Toxicology and Applied Pharmacology*.

[19] Oyoshi M. K., He R., Li Y. (2012). Leukotriene B4-driven neutrophil recruitment to the skin is essential for allergic skin inflammation. *Immunity*.

[20] Yokomizo T. (2015). Two distinct leukotriene B4 receptors, BLT1 and BLT2. *Journal of Biochemistry*.

[21] McIlroy A., Caron G., Blanchard S. (2006). Histamine and prostaglandin E2 up-regulate the production of Th2-attracting chemokines (CCL17 and CCL22) and down-regulate IFN-*γ*-induced CXCL10 production by immature human dendritic cells. *Immunology*.

[22] Bruckner M., Dickel D., Singer E., Legler D. F. (2012). Distinct modulation of chemokine expression patterns in human monocyte-derived dendritic cells by prostaglandin E_2_. *Cellular Immunology*.

[23] Lee C. W., Lin Z. C., Hu S. C. (2016). Urban particulate matter down-regulates filaggrin via COX2 expression/PGE2 production leading to skin barrier dysfunction. *Scientific Reports*.

[24] Jin H., He R., Oyoshi M., Geha R. S. (2009). Animal models of atopic dermatitis. *The Journal of Investigative Dermatology*.

[25] Hirai H., Tanaka K., Yoshie O. (2001). Prostaglandin D2 selectively induces chemotaxis in T helper type 2 cells, eosinophils, and basophils via seven-transmembrane receptor CRTH2. *The Journal of Experimental Medicine*.

[26] Takeda K., Osakabe A., Saito S. (2005). Components of protocyanin, a blue pigment from the blue flowers of Centaurea cyanus. *Phytochemistry*.

[27] Sarker S. D., Savchenko T., Whiting P., Šik V., Dinan L. N. (1997). Moschamine, CIS-moschamine, moschamindole and moschamindolol: four novel indole alkaloids from Centaurea moschata. *Natural Product Letters*.

[28] Sarker S. D., Laird A., Nahar L., Kumarasamy Y., Jaspars M. (2001). Indole alkaloids from the seeds of Centaurea cyanus (Asteraceae). *Phytochemistry*.

[29] Bernard P., Humbert F., Do Q. T. (2015). *Compositions comprising an extract of cornflower for cosmetic and pharmaceutical uses*.

[30] Abboud D., Daubeuf F., Do Q. T. (2015). A strategy to discover decoy chemokine ligands with an anti-inflammatory activity. *Scientific Reports*.

[31] https://www.tocris.com/products/c-021-dihydrochloride_3581

[32] Orlando B. J., Malkowski M. G. (2016). Crystal structure of rofecoxib bound to human cyclooxygenase-2. *Acta Crystallographica Section F Structural Biology Communications*.

[33] Madhavi Sastry G., Adzhigirey M., Day T., Annabhimoju R., Sherman W. (2013). Protein and ligand preparation: parameters, protocols, and influence on virtual screening enrichments. *Journal of Computer-Aided Molecular Design*.

[34] Harder E., Damm W., Maple J. (2016). OPLS3: a force field providing broad coverage of drug-like small molecules and proteins. *Journal of Chemical Theory and Computation*.

[35] Halgren T. A., Murphy R. B., Friesner R. A. (2004). Glide: a new approach for rapid, accurate docking and scoring. 2. Enrichment factors in database screening. *Journal of Medicinal Chemistry*.

[36] Sherman W., Day T., Jacobson M. P., Friesner R. A., Farid R. (2006). Novel procedure for modeling ligand/receptor induced fit effects. *Journal of Medicinal Chemistry*.

[37] Fujisawa T., Fujisawa R., Kato Y. (2002). Presence of high contents of thymus and activation-regulated chemokine in platelets and elevated plasma levels of thymus and activation-regulated chemokine and macrophage-derived chemokine in patients with atopic dermatitis. *The Journal of Allergy and Clinical Immunology*.

[38] Kakinuma T., Nakamura K., Wakugawa M. (2001). Thymus and activation-regulated chemokine in atopic dermatitis: serum thymus and activation-regulated chemokine level is closely related with disease activity. *The Journal of Allergy and Clinical Immunology*.

[39] Matsuo K., Nagakubo D., Komori Y. (2018). CCR4 is critically involved in skin allergic inflammation of BALB/c mice. *The Journal of Investigative Dermatology*.

[40] Matsuo K., Hatanaka S., Kimura Y. (2019). A CCR4 antagonist ameliorates atopic dermatitis-like skin lesions induced by dibutyl phthalate and a hydrogel patch containing ovalbumin. *Biomedicine & Pharmacotherapy*.

[41] Kenakin T. (2005). New concepts in drug discovery: collateral efficacy and permissive antagonism. *Nature Reviews. Drug Discovery*.

[42] Ajram L., Begg M., Slack R. (2014). Internalization of the chemokine receptor CCR4 can be evoked by orthosteric and allosteric receptor antagonists. *European Journal of Pharmacology*.

[43] Déruaz M., Bonvin P., Severin I. C. (2013). Evasin-4, a tick-derived chemokine-binding protein with broad selectivity can be modified for use in preclinical disease models. *The FEBS Journal*.

[44] Webb L. M., Smith V. P., Alcami A. (2004). The gammaherpesvirus chemokine binding protein can inhibit the interaction of chemokines with glycosaminoglycans. *The FASEB Journal*.

[45] Denisov S. S., Ramirez-Escudero M., Heinzmann A. C. A. (2020). Structural characterization of anti-CCL5 activity of the tick salivary protein evasin-4. *The Journal of Biological Chemistry*.

[46] Fulle L., Steiner N., Funke M. (2018). RNA aptamers recognizing murine CCL17 inhibit T cell chemotaxis and reduce contact hypersensitivity in vivo. *Molecular Therapy*.

[47] Regenass P., Abboud D., Daubeuf F. (2018). Discovery of a locally and orally active CXCL12 neutraligand (LIT-927) with anti-inflammatory effect in a murine model of allergic airway hypereosinophilia. *Journal of Medicinal Chemistry*.

[48] Steurer M., Montillo M., Scarfo L. (2019). Olaptesed pegol (NOX-A12) with bendamustine and rituximab: a phase IIa study in patients with relapsed/refractory chronic lymphocytic leukemia. *Haematologica*.

[49] Kumarasamy Y., Middleton M., Reid R. G., Nahar L., Sarker S. D. (2003). Biological activity of serotonin conjugates from the seeds of Centaurea nigra. *Fitoterapia*.

[50] Hodaj E., Tsiftsoglou O., Abazi S., Hadjipavlou-Litina D., Lazari D. (2017). Lignans and indole alkaloids from the seeds of Centaurea vlachorum Hartvig (Asteraceae), growing wild in Albania and their biological activity. *Natural Product Research*.

[51] Liang N., Kitts D. D. (2016). Role of chlorogenic acids in controlling oxidative and inflammatory stress conditions. *Nutrients*.

[52] Hou D. D., Zhang W., Gao Y. L. (2019). Anti-inflammatory effects of quercetin in a mouse model of MC903-induced atopic dermatitis. *International Immunopharmacology*.

[53] Caglayan Sozmen S., Karaman M., Cilaker Micili S. (2016). Resveratrol ameliorates 2,4-dinitrofluorobenzene-induced atopic dermatitis-like lesions through effects on the epithelium. *PeerJ*.

[54] Aral M., Arican O., Gul M. (2006). The Relationship Between Serum Levels of Total IgE, IL-18, IL-12, IFN-*γ* and Disease Severity in Children With Atopic Dermatitis. *Mediators of Inflammation*.

[55] Kagami S., Kakinuma T., Saeki H. (2003). Significant elevation of serum levels of eotaxin-3/CCL26, but not of eotaxin-2/CCL24, in patients with atopic dermatitis: serum eotaxin-3/CCL26 levels reflect the disease activity of atopic dermatitis. *Clinical and Experimental Immunology*.

[56] Canavese M., Altruda F., Silengo L. (2010). Therapeutic efficacy and immunological response of CCL5 antagonists in models of contact skin reaction. *PLoS One*.

[57] Noma N., Asagiri M., Takeiri M. (2015). Inhibition of MMP-2-mediated mast cell invasion by NF-kappaB inhibitor DHMEQ in mast cells. *International Archives of Allergy and Immunology*.

[58] Kawashima S., Hayashi M., Takii T. (1998). Serotonin derivative, N-(p-coumaroyl) serotonin, inhibits the production of TNF-alpha, IL-1alpha, IL-1beta, and IL-6 by endotoxin-stimulated human blood monocytes. *Journal of Interferon & Cytokine Research*.

[59] Takii T., Kawashima S., Chiba T. (2003). Multiple mechanisms involved in the inhibition of proinflammatory cytokine production from human monocytes by N-(p-coumaroyl)serotonin and its derivatives. *International Immunopharmacology*.

[60] Tsai A., Kulmacz R. J. (2000). Tyrosyl radicals in prostaglandin H synthase-1 and -2. *Prostaglandins & Other Lipid Mediators*.

[61] Park J. B. (2012). Synthesis, biological activities and bioavailability of moschamine, a safflomide-type phenylpropenoic acid amide found in Centaurea cyanus. *Natural Product Research*.

[62] Jo A.-r., Han H.-s., Seo S. (2017). Inhibitory effect of moschamine isolated from _Carthamus tinctorius_ on LPS- induced inflammatory mediators via AP-1 and STAT1/3 inactivation in RAW 264.7 macrophages. *Bioorganic & Medicinal Chemistry Letters*.

[63] Linnet J., Jemec G. B. (2001). Anxiety level and severity of skin condition predicts outcome of psychotherapy in atopic dermatitis patients. *International Journal of Dermatology*.

[64] Kawana S., Kato Y., Omi T. (2010). Efficacy of a 5-HT1a receptor agonist in atopic dermatitis. *Clinical and Experimental Dermatology*.

